# Efficacy of the combination of long-acting release octreotide and tamoxifen in patients with advanced hepatocellular carcinoma: a randomised multicentre phase III study

**DOI:** 10.1038/sj.bjc.6603901

**Published:** 2007-08-07

**Authors:** G Verset, C Verslype, H Reynaert, I Borbath, P Langlet, A Vandebroek, M Peeters, G Houbiers, S Francque, M Arvanitakis, J-L Van Laethem

**Affiliations:** 1 Erasme University Hospital, ULB, Brussels, Belgium; 2 University Hospital Gasthuisberg, KUL, Leuven, Belgium; 3 University Hospital AZ-VUB, Jette, Belgium; 4 Saint-Luc University Hospital, Brussels, Belgium; 5 University Hospital Brugmann, Brussels, Belgium; 6 AZ Middelheim, Antwerpen, Belgium; 7 University Hospital Gent, Gent, Belgium; 8 CHBA Seraing, Seraing, Belgium; 9 University Hospital Antwerpen, Edegem-Antwerpen, Belgium

**Keywords:** hepatocellular carcinoma (HCC), somatostatin analogues, octreotide, tamoxifen

## Abstract

To assess the efficacy of the combination of long-acting release (LAR) octreotide and tamoxifen (TMX) for the treatment of advanced hepatocellular carcinoma (HCC). A total of 109 patients with advanced HCC were randomised to receive octreotide LAR combined with TMX (*n*=56) (experimental treatment group) or TMX alone (*n*=53; control group). The clinical, biological and tumoural parameters were recorded every 3 months until death. Primary end point was patient survival; secondary end points were the impact of therapy on tumour response, quality of life and variceal bleeding episodes. Univariate and multivariate analyses were performed for assessment of specific prognostic factors. The median survival was 3 months (95% CI 1.4–4.6) for the experimental treatment group and 6 months (CI 95% 2–10) for the control group (*P*=0.609). There was no difference in terms of *α*-foetoprotein (*α*-FP) decrease, tumour regression, improvement of quality of life and prevention of variceal bleeding between the two groups. Variables associated with a better survival in the multivariate analysis were: presence of cirrhosis, *α*-FP level <400 ng ml^−1^ and Okuda stage I. The combination of octreotide LAR and TMX does not influence survival, tumour progression or quality of life in patients with advanced HCC.

Hepatocellular carcinoma (HCC) is the fifth most common cancer in the world and the third most common cause of cancer mortality. Its incidence is increasing worldwide because of the dissemination of hepatitis B and C virus infection (Van Vlierberghe *et al*, 2004).

The characteristics of HCC vary geographically. In Asia and sub-Saharan Africa, HCC occurs mainly in young patients without cirrhosis, infected early in life by hepatitis B virus. By contrast, in Western countries, HCC develops in older patients with alcoholic or hepatitis C-related cirrhosis. Curative therapeutic options usually consist of transplantation or resection, but for most cases, palliative therapy is the only option (Llovet and Beaugrand, 2003).

In this setting, best supportive care (BSC) including regulation of fluid retention with diuretics, abdominal paracentesis and albumin infusion, and prevention of variceal bleeding with *β*-blockers is the only recommendable standard medical therapy. So far, the majority of medical treatments (apart BSC) assessed in advanced HCC have shown disappointing results and do not significantly change the natural course of the disease. The role of trans-arterial chemo-embolisation in the management of advanced HCC remains questionable, although there are some studies reporting survival benefit (Llovet *et al*, 2002; Lo *et al*, 2002; Groupe d'Etude et de Traitement du Carcinome Hepatocellulaire, 2004). Chemotherapeutic options have also been reported as disappointing and hazardous in the management of HCC due to the impaired hepatic function and the relative chemoresistance (Nowak *et al*, 2004). Owing to its anti-angiogenic and presumably antiproliferative properties, interferon-*α* has been used to treat advanced HCC but failed to be efficient in improving survival (Llovet *et al*, 2000).

Regarding hormonal therapy, it clearly appears that flutamide or LH–RH agonists are not effective (Manesis *et al*, 1995; Groupe d'Etude et de Traitement du Carcinome Hepatocellulaire, 2004). Oestrogen receptors are present in 33% of HCC cases and anti-oestrogen therapy using tamoxifen (TMX) was widely evaluated in HCC leading to discrepant results regarding its efficacy (Manesis *et al*, 1995; CLIP group, 1998; Mathurin *et al*, 1998; Liu *et al*, 2000). While the former meta-analysis suggested a possible beneficial role, a recently published meta-analysis reported no significant benefit with TMX alone and overall, its role as single hormonal agent in the management of advanced HCC was therefore claimed to be unbeneficial despite some recent positive results in subgroups of patients (Barbare *et al*, 2005; Nowak *et al*, 2005).

Somatostatin is a hormone with well-proven efficacy for the treatment of hormone-producing tumours, mainly neuroendocrine tumours. The biological effects of somatostatin are mediated through five G-protein-coupled receptors (SSTR1-5). Similarly to neuroendocrine tumours, it has been proven that some solid cancers, including HCC, also express SSTRs (Reubi *et al*, 1999; Bläker *et al*, 2004). These data were recently confirmed by a study that investigated expression of SSTRs in human HCC using immunohistochemistry and reverse transcription-PCR (Reynaert *et al*, 2004). An *in vitro* activity of lanreotide, a long-acting somatostatin analogue, has been demonstrated on hepatoma cell culture (Hep G_2_ cell) consisting in decreased cellular proliferation (S-phase) and increased apoptosis, suggesting thus an antiproliferative effect of somatostatin analogues (Raderer *et al*, 2000). For the other somatostatin analogue, octreotide, an antiproliferative effect on tumour growth in nude mice bearing HCC xenografts has also been recently demonstrated (Jia *et al*, 2003). Kouroumalis *et al* (1998) reported the first randomised clinical trial assessing octreotide in advanced HCC and showing a significant impact on patient survival. Since this study, confirmation data are lacking, as the results from recent randomised studies comparing octreotide long-acting release (LAR) *vs* BSC or placebo failed to show a survival benefit (Yuen *et al*, 2002; Barbare *et al*, 2005; Becker *et al*, 2007).

In the present study, we aimed to evaluate an hormonal combined therapy of octreotide and TMX based on the following; several data have suggested that the deregulation of the insulin-like growth factor axis (interaction of insulin-growth factor (IGF)-I and -II with the IGF-1R) plays a pivotal role in hepatocarcinogenesis and that the inhibition of the IGF-/IGFR-signalling system could be a valuable and promising approach of HCC treatment; it was suggested that octreotide is able to inhibit this pathway (Ren *et al*, 1992; Scharf *et al*, 2001). Somatostatin analogues have been reported to decrease IGF-I levels and receptor expression; furthermore, octreotide has been combined with TMX in preclinical and clinical settings and in a rat model, this combination was able to decrease IGF-I serum levels and hepatic gene expression more profoundly than either agent administered alone (Huynh and Pollak, 1994; Weckbecker *et al*, 1994; Pollak, 1996; Bontenbal *et al*, 1998). In patients with breast cancer, such a combination has yielded a more profound suppression of IGF-I level, which was however not translated into a clinical benefit (Ingle *et al*, 1999; Helle *et al*, 2005). On the basis of this possible synergy between two hormonal therapies, we designed this prospective randomised multicentre study aiming to evaluate the efficacy of the doublet octreotide+TMX for the treatment of advanced HCC, not amenable to surgery or local therapy. In the control arm, we chose to give TMX since its role was not yet definitely clarified at the start of the current study in 2001, the aim of the study being to demonstrate an advantage of administering two hormonal therapies on a single one. Primary end point was survival; secondary end points were the impact of therapy on tumour response, quality of life and also on the incidence of variceal bleeding, as somatostatin analogues are able to decrease portal pressure (de Franchis, 2000; Spahr *et al*, 2007).

## PATIENTS AND METHODS

### Study design and admission criteria

Between March 2001 and March 2005, 18 Belgian Hospital Centers included patients with advanced HCC in the study. Written informed consent was obtained from each patient before entering the study, and the study protocol was approved by the ethics committees of all participating centres.

Patients were eligible to be admitted to the study if they fulfilled the following criteria: (1) diagnosis of HCC established by histology or by the coincidental findings of two imaging techniques showing a nodule of >2 cm with arterial hypervascularisation, or by a single positive imaging technique associated with *α*-foetoprotein (*α*-FP) level of >400 ng ml^−1^ (according to the Barcelona-2000 EASL conference; Bruix *et al*, 2001) (2) advanced disease not amenable to curative resection, liver transplant or locoregional treatment after multidisciplinary conference (patients with recurrence after surgery/transplantation were eligible), (3) age above 18 years, (4) Karnofsky performance status (KPS) ⩾60, (5) life expectancy of at least 2 months.

Exclusion criteria were: (1) history of other malignancy, (2) unstable serious coexisting medical condition, (3) known cerebral metastasis, (4) Child–Pugh score >10, (5) platelet count <20 000 mm^−3^.

Baseline assessment included a complete physical examination, determination of KPS, laboratory tests and tumour characteristics assessed by imaging and Okuda score (Okuda *et al*, 1985). The laboratory tests consisted of complete blood count, prothrombin time, liver biochemistry, serum albumin level (to determine the Child–Pugh score) and *α*-FP level.

### Interventions and randomisation

Patients were stratified by centres and Child–Pugh score, and were assigned through a computer-generated randomisation list to the experimental treatment group (octreotide+TMX) or to the control group (TMX alone) in 1 : 1 ratio. An intramuscular injection of 30 mg octreotide LAR was administered once monthly in the experimental treatment group, and a daily dose of 20 mg TMX was administered orally in both groups. Best supportive care including *β*-blockers, diuretics, paracentesis and albumin infusion was allowed in both arms.

### Follow-up and end points

Patients were followed up monthly until death. All the clinical (included KPS) and biological parameters and all drugs administered were recorded each month. Any treatment-related adverse events was monitored and noted. In addition, the number of variceal bleeding episodes was recorded in each patient from both groups.

The tumour response was assessed using the serum *α*-FP level and the sum of longest diameter of the target lesions measured at baseline, 3 and 12 months according to the RECIST criteria (Duffaud and Therasse, 2000).

Radiological complete response was defined as complete disappearance of the tumour; partial response as at least a 30% decrease in the diameter of the target lesion without simultaneous increase in the size of any other lesion or appearance of new lesion; progressive disease as at least a 20% increase in the diameter of the target lesion or emergence of new lesions; stable disease for the intermediary cases.

Similarly, an increase or a decrease of the *α*-FP level was defined by a variation of more than 25% compared to the baseline value.

Survival was calculated from the date of randomisation to the date of last follow-up or death.

The primary end point of the study was the effect of treatment on survival; secondary end points were the effects on tumour progression, quality of life and variceal bleeding.

### Sample size calculation and statistical analysis

On the basis of the published data, 1-year survival in the control group ranges from 10 to 30%. Expecting a 30% 1-year survival rate in the control group, aiming to achieve a 50% 1-year survival rate in the experimental treatment group, and requiring a level of statistical significance of 0.05 and a power of 0.9, 90 patients were planned to be included in the study (45 in each arm).

Continuous variables were expressed in median with minimal and maximal value, and in percentages for discretes variables. The Mann–Whitney test was used to compare continuous variables and the *χ*^2^ test or Fischer's exact test for the discrete variables.

Survival curves were drawn according to the Kaplan–Meier method and compared by the log-rank test. Survival was analysed on an intention-to-treat (ITT) basis; an exploratory subgroup analysis was also performed only considering the patients with treatment exposure ⩾2 months.

A stratified Cox proportional hazard regression model was used for multivariate analysis, using age, gender, treatment by octreotide LAR, presence of cirrhosis, Child–Pugh stage, *α*-FP level and Okuda stage as covariates, to determine independent prognostics factors possibly affecting survival.

*P*-values <0.05 were considered as statistically significant. Data analysis was carried out with the computer programme SPSS version 11 (SPSS Inc., Chicago, IL, USA).

## RESULTS

### Baseline characteristics

**Figure 1:**
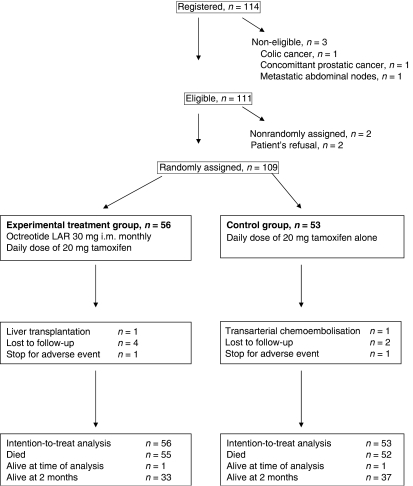
Participant flow chart.

One-hundred and fourteen patients were included between March 2001 and March 2005. Three patients were found ineligible after randomisation because a different tumour was diagnosed: one with prostatic adenocarcinoma, one with colonic adenocarcinoma and one with peritoneal carcinomatosis from an adenocarcinoma. Two patients decided to discontinue the study a few days after randomisation without receiving any treatment (Figure 1).

One-hundred and nine patients were thus analysed: 56 were assigned to the experimental treatment group and 53 to the control group. Groups were comparable with respect to age, gender, presence of cirrhosis, Child–Pugh score, aetiology of hepatopathy, Okuda classification, KPS, *α*-FP level and size of target lesion (Table 1).

The median follow-up in this study was 3 months (range, 0.1–31), and the median number of octreotide LAR injections was 2 (range, 1–31) in the octreotide group. All patients continued to take TMX until death.

### Adverse events and reasons of study discontinuation

Four patients discontinued the study for drug-related toxicity. Only one patient in the experimental treatment group stopped the treatment for diarrhoea (Table 2).

### Survival data

**Figure 2:**
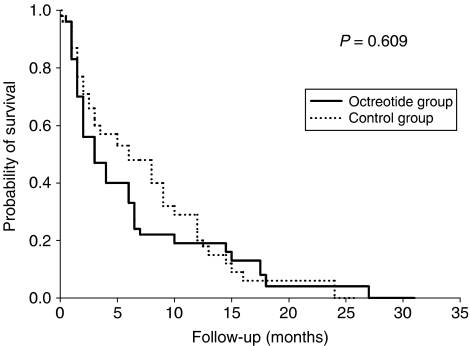
Survival curves for experimental treatment (octreotide+TMX) and control (TMX) groups for 109 patients (ITT analysis). Cumulative survival probability is given on the *y* axis and time in months is given on the *x* axis. Log-rank test showed no significant differences between survival for the experimental treatment group (solid line, *n*=56) and the control group (dashed line, *n*=53).

In ITT analysis, the median survival was 3 months (95% CI 1.4–4.5) for the experimental treatment group and 6 months (95% CI 2–10) for the control group (*P*=0.609) (Figure 2). Among the 70 patients who had a treatment exposure of ⩾2 months, there was no difference in survival between the two groups (8 months (95% CI 5.6–10.4) for the experimental treatment group and 6 months (95% CI 4.1–7.9) for the control group (*P*=0.930).

### Tumour response, quality of life and variceal bleeding

Tumour response at 3 months was assessed by the evolution of target lesion's diameter and *α*-FP level. No significant difference was observed between the two groups (Table 3).

Evolution of KPS at 3 months was also studied to evaluate a possible impact of octreotide on quality of life. The median KPS after 3 months on therapy was similar: 80 in each group (95% CI 40–100 octreotide group, 30–100 for control group; *P*=0.769; Table 3).

In addition, there was no difference in terms of variceal bleeding episodes between the two groups (0 bleeding in the experimental treatment group *vs* 2 bleeding in the control group; *P*=0.477).

### Prognostic factors of survival

The univariate analysis showed a significant relation between survival and Okuda stage, *α*-FP level, Child–Pugh score and absence of underlying cirrhosis (Table 4).

In multivariate analysis, absence of underlying cirrhosis, *α*-FP level and Okuda stage were revealed as independent prognostic factors affecting survival (Table 5). Patients with a HCC in a non-cirrhotic liver deceased significantly earlier than patients with cirrhosis: relative risk (RR)=2.002 (CI 95% 1.044–4.073). Similarly, *α*-FP level>400 ng ml^−1^ and Okuda stage III have negative impact on overall survival: RR=2.810 (95% CI 1.469–5.374) and RR=3.014 (95% CI 1.118–8.126) respectively.

## DISCUSSION

Most of the patients with HCC are not eligible for surgical or locoregional procedures (trans-arterial chemo-embolisation, radiofrequency ablation, percutaneous ethanol injection). For these advanced cases, various systemic treatments have been proposed, poorly or not impacting on survival (Bruix *et al*, 2001).

Systemic chemotherapy and immunotherapy (IFN-*α*) have only marginal activity and are poorly tolerated in cirrhotic patients (Llovet *et al*, 2000; Nowak *et al*, 2004).

Since the Kouroumali's publication on the impact of somatostatin analogues in advanced HCC, results of the three subsequent randomised controlled trials assessing octreotide LAR were disappointing, reporting only very poor survival data and benefit and infirming the initial study (Table 6).

The first trial, published in 1998 by Kouroumalis has shown a significant improvement on survival and on quality of life in 28 patients with advanced HCC treated with subcutaneous octreotide compared with the control group (*n*=30); afterwards, three other randomised studies using octreotide LAR did not confirm these results (Yuen *et al*, 2002; Barbare *et al*, 2005; Becker *et al*, 2007). The first study using octreotide LAR, reported by Yuen *et al* (2002), was controversial and criticable because the selected patients had a very short survival (1.9 months in the control group (*n*=35) *vs* 2 months in the octreotide group (*n*=35)), probably due to a high proportion of advanced HCC with CLIP score >4 and a poor treatment exposure. In this setting, it was impossible to detect any benefit for octreotide treatment. The two other negative randomised studies with octreotide LAR reported by Barbare *et al* (2005) and Becker *et al* (2007) were more relevant because they used a placebo control arm; the tumour characteristics were well described and the patients' cohorts were larger. Survival in the two groups was longer but not significantly different. Several additional smaller non-randomised studies were also published reporting conflicting or inconclusive results that do not help us very much (Raderer *et al*, 2000; Dimitroulopoulos *et al*, 2002; Rabe *et al*, 2002; Samonakis *et al*, 2002).

Our study conclusions are on the same line as the others mentioned above, reporting similar data and proportion of advanced disease (18% of Okuda III) that German Study did (Becker *et al*, 2007).

However, when we designed the study in 2001, the real impact of blocking oestrogen receptors by TMX in HCC was still controversial and the unique available meta-analysis suggested at this time a small advantage for this therapy (Mathurin *et al*, 1998). On the other hand, we found a rationale for using a synergistic combination of two hormonal therapies as it was shown in preclinical studies and metastatic breast cancer (Huynh and Pollak, 1994; Ingle *et al*, 1999; Helle *et al*, 2005). Furthermore, it is now clear that IGF-/IGFR-signalling pathway plays a pivotal role in hepatocarcinogenesis (Scharf *et al*, 2001). Octreotide is a good candidate for inhibiting the IGF axis, and a synergistic effect with TMX has been demonstrated in animal models (Huynh and Pollak, 1994). This is why we preferentially choose to compare this experimental doublet therapy to a control one based on a single anti-hormonal agent, in the absence of other standard and effective therapeutic options. As this combination was not able to demonstrate any survival benefit in the experimental group, we did not evaluate the modulation of the IGF axis in the present study; however, we strongly believe that more potent IGF-R inhibition using new targeted agents, such as NVP-AEW541 deserves further exploration in HCC (Hopfner *et al*, 2006).

Interestingly, we observed no survival benefit using this doublet, even in the patients exposed to the drugs more than 2 months. This is a remarkable finding while in the previous Korean study, a major criticism was that the therapeutic intervention was very short and survival extremely limited (Yuen *et al*, 2002).

No difference was observed between the two groups in terms of radiological tumour response, decrease in *α*-FP levels and impact on quality of life. Moreover, we failed to observe an effect of preventing variceal bleeding, despite the hypothesis of a potential reduction in hepatic venous pressure gradient induced by octreotide, recently confirmed by a randomised controlled study comparing octreotide LAR to placebo (Spahr *et al*, 2007).

In the univariate analysis, we identified the following known factors of poor prognostic: *α*-FP ⩾400 ng ml^−1^, Okuda stage III and the severity of cirrhosis assessed by the Child–Pugh score, as observed in the German study (Becker *et al*, 2007). In the multivariate analysis, *α*-FP level and the Okuda stage persisted as independent variables. More surprisingly, the absence of cirrhosis emerged as a negative predictor in univariate and multivariate analysis. This could be explained by the fact that the mechanisms of hepatocarcinogenesis and the characteristics of the tumour in non-cirrhotic liver are different and could confer a more aggressive behaviour of the HCC. Another explanation could be a selection bias because we only assessed the tumour characteristics by *α*-FP level and the Okuda stage without evaluation of tumour morphology, vascular invasion and the existence of distant metastasis. It is therefore possible that, in non-cirrhotic patients, HCC was diagnosed at a more advanced stage with portal invasion or secondary localisation, contrasting with cirrhotic patients who benefit from screening programmes and earlier diagnosis.

Several arguments could be mentioned to explain the negative results of the study. First, an imbalance regarding vascular involvement and extra-hepatic metastases could exist between the experimental treatment and the control groups, as we did not assess these parameters in our trial. Nevertheless, in the multicentre French and German studies (Barbare *et al*, 2005; Becker *et al*, 2007), these two parameters were included and, clearly, this did not result in a benefit for octreotide LAR in the reported analysis. Secondly, somatostatin acts through five G-protein-coupled receptors (SSTR 1–5). These SSTRs have been identified in various solid tumours including human HCC. It seems that only 40% of HCC expressed SSTRs and that the expression pattern and the expression levels of SSTRs are heterogeneous in HCCs and are independent of the stage, the histological type of the tumour or the underlying liver disease.

SSTR5 is the most important subtype of receptor expressed by HCC, followed by SSTR3, SSTR1 and SSTR2. Pharmacological studies have shown that the long-acting somatostatin analogues have high affinity for SSTR2, an intermediate affinity for SSTR5 and SSTR3, and almost no affinity for SSTR1 and SSTR4 (affinity rank order: SSTR2>SSTR5>SSTR3) (Reubi *et al*, 1999; Bläker *et al*, 2004; Reynaert *et al*, 2004).

Animal models and *in vitro* studies have shown that octreotide is a potent anti-angiogenic factor. This effect is probably mediated by SSTR3 and is dose-dependent (Jia *et al*, 2003). It is therefore possible that the positive results observed in the Greek study using short-acting somatostatin analogues may be explained, at least in part, by its suppressive effects on angiogenesis and therefore on tumour blood supply. This effect may not be observed in studies using long-acting analogues due to pharmacological reasons.

In conclusion, our results do not support a synergistic effect of the combination of octreotide and TMX as suggested in breast cancers and reinforce the lack of efficacy for hormonal therapy in HCC, even for combination.

In this study, octreotide LAR associated with TMX did not provide any benefit in terms of survival, tumour response or quality of life compared with TMX alone in patients with advanced HCC. Hence, octreotide LAR is not recommendable for this indication, outside of clinical trials including patients with advanced HCC specifically expressing SSTRs. Further studies are therefore mandatory to detect subgroups of patients with HCC that could benefit from treatment with somatostatin analogues.

## Figures and Tables

**Table 1:** Baseline characteristics

	**Experimental treatment group (*n*=56)**	**Control group (*n*=53)**	***P***
Median age (years) (range)	64.5 (39–84)	68 (38–83)	0.202
Male gender (%)	78.6	83	0.366
Histology proven (%)	55.4	71.7	0.058
Cirrhosis (%)	82	79.2	0.298
			
*Child* (%)			
A	55.4	71.7	0.208
B	39.3	24.5	
C	5.4	3.8	
			
*Aetiology* (%)			
OH	52.4	40.5	0.742
HBV	9.5	10.8	
HCV	33.3	40.5	
Other	4.8	8.1	
Median KPS (range)	80 (60–100)	80 (60–100)	0.373
Median *α*-foetoprotein (ng ml^−1^) (range)	265 (1.6–498000)	98.1 (0–1135659)	0.378
Median diameter of target lesion (mm) (range)^a^	55 (3–160)	56 (4.1–280)	0.823
			
*Okuda stage* (%)			
1	36.4	41.3	0.565
2	50.9	52.2	
3	12.7	6.5	

HBV=hepatitis B virus; HCV=hepatitis C virus; KPS=Karnofsky performance status; OH=ethanol.

a Measurable for 100 patients (51: experimental treatment group, 49: control group).

**Table 2:** Reasons for study discontinuation and study treatment-related adverse events

**Causes**	**Experimental treatment group (*n*=56)**	**Control group (*n*=53)**
General status degradation	6	5
Non-compliance/LFU	6	3
		
*Adverse event* ⩾ *grade 3*	1	3
Diarrhoea grade 3	1	
Nausea grade 3		1
Flush		1
Gynaecomastia		1
		
*Other treatments*	2	2
Transplantation/surgery	2	
TACE		1
PEI		1

LFU=lost of follow-up; PEI=percutaneous ethanol injection; TACE=transarterial chemoembolisation.

**Table 3:** Assessment of tumour response and quality of life at 3 months

**Parameters**	**Experimental treatment group (*n*=25)**	**Control group (*n*=30)**	***P***
*Radiological response* a			
PR(%)	5	3.7	0.872
Progression (%)	65	59.3	
Stable (%)	30	37	
			
α*-Foetoprotein level*^b^			
Decrease >25% (%)	27.8	10.7	0.282
Stable (%)	16.7	28.6	
Increase >25% (%)	55.6	60.7	
			
Median KPS	80	80	0.769

a Data available for 20 patients in the experimental treatment group and 26 in the control group.

b Data available for 18 patients in the experimental treatment group and 28 in the control group.

PR=partial response; KPS=Karnofsky performance status.

**Table 4:** Variables associated with better survival in univariate analysis

**Baseline parameters**	**Median survival**	**95% CI**	***P***
*Age* (*years*)			
⩽70	4.5	1.5–7.5	0.250
>70	4.0	1.8–6.2	
			
*Gender*			
Male	6.0	3.1–8.9	0.139
Female	2.0	1.0–3.0	
			
*Treatment*			
Experimental (octreotide+TMX)	3.0	0.9–5.1	0.574
Control (TMX alone)	6.0	1.9–10.1	
			
			
*Cirrhosis*			
Yes	6.0	2.8–9.2	0.049
No	3.0	0.6–5.4	
			
*Child*			
A	6.0	4.3–7.7	0.031
B	1.5	0.7–2.3	
C	2.5	0.1–4.9	
			
*α*-*Foetoprotein* (*ng* *ml*^−*1*^)			
<400	6.0	2.7–9.3	0.036
⩾400	2.0	1.1–2.9	
			
*Okuda stage*			
I	7.0	1.8–12.2	0.01
II	3.0	1.5–4.5	
III	1.5	1.0–2.0	

**Table 5:** Variables associated with a better survival in multivariate analysis

	**RR**	**95% CI**	***P***
*Cirrhosis*			
Yes	1		
No	2.002	1.044–4.073	**0.037**
			
*α*-*foetoprotein*			
<400 ng ml^−1^	1		
⩾400 ng ml^−1^	2.810	1.469–5.374	**0.002**
			
*Okuda stage*			
I	1		
II	1.154	0.550–2.422	0.705
III	3.014	1.118–8.126	**0.029**

95% CI=95% confidence interval; RR=relative risk.

Bold values are statistically significant.

**Table 6:** Randomised trials evaluating the efficacy of octreotide in HCC

**Authors**	**Treatment**	***n***	**Median survival (month)**	***P***
Kouroumalis *et al* (1998)	Octreotide	28	13	0.002
	BSC	30	4	
Yuen *et al* (2002)	Octreotide LAR	35	1.9	NS
	BSC	35	2	
Barbare *et al* (2005)	Octreotide LAR	133	6.77	NS
(Abstract)	Placebo	131	7.86	
Becker *et al* (2007)	Octreotide LAR	60	4.7	NS
	Placebo	59	5.3	
Present study	Octreotide LAR+TMX	57	3.0	NS
	TMX	59	5.3	
